# Using Geographic Information Systems (GIS) for Targeted National Recruitment of Community-Dwelling Caregivers Managing Dementia-Related Behavioral and Psychological Symptoms: A Recruitment Approach for a Randomized Clinical Trial

**DOI:** 10.4236/jgis.2021.133017

**Published:** 2021-05-12

**Authors:** Danny L. Scerpella, Nicole G. Bouranis, Melinda J. Webster, Maria Dellapina, Sokha Koeuth, Lauren J. Parker, Helen C. Kales, Laura N. Gitlin

**Affiliations:** 1 School of Public Health, Johns Hopkins University, Baltimore, USA; 2 College of Nursing and Health Professions, Drexel University, Philadelphia, USA; 3 Department of Psychiatry, University of California-Davis, Sacramento, USA

**Keywords:** Geographic Information Systems, Recruitment, Clinical Trial, GIS, Dementia or Caregiving

## Abstract

Over 16 million caregivers of people living with dementia require support in a range of issues, including self-care, disease education, and guidance for how to manage behavioral and psychological symptoms of dementia (BPSD). Non-pharmacological interventions are needed to address these areas, and online applications have been shown to be safe and effective. To ensure the efficacy of such interventions, racially, ethnically, geographically, and socioeconomically diverse participants must be recruited to increase the generalizability of study outcomes. This protocol paper describes a recruitment plan using Geographic Information Systems (GIS) to reach a representative sample of caregivers across the United States for a national Phase III clinical study. Using publicly available census data from the American Community Survey (ACS), combined with location data for local aging resources such as Area Agencies on Aging (AAA), recruitment will be derived from data analysis conducted in ESRI ArcGIS v10.7.1. Datasets including age, gender, income, and education will be assessed nationally at the county and census tract spatial scale in a nine-step process to develop recruitment priority areas containing high concentrations of eligible participants living in the community. Overall, the current protocol will demonstrate the value of GIS in tailoring targeted outreach strategies to recruit community-dwelling populations through local resource institutions. This novel approach may have far-reaching implications in future recruitment initiatives and help to secure racially/ethnically diverse samples.

## 1. Background

Over 16 million caregivers in the United States provide 257 billion dollars worth of unpaid care to people living with dementia [1]. Behavioral and psychological symptoms of dementia (BPSD), including agitation, apathy, rejection of care, shadowing, depression, executive dysfunction, sleep disturbances and psychosis are common hallmarks of the disease, regardless of etiology [2] [3]. Of these various behaviors that occur throughout the disease life course, how they manifest is unique to each person living with dementia. Furthermore, behavioral and psychological symptoms are typically under-detected and under-treated and families are left on their own to manage. As such, individuals caring for people living with dementia need customized support when managing complex dementia-related behavioral scenarios [4].

Since dementia-related burdens are an increasingly prevalent concern, it is vital to recognize the impact they have in decreasing quality of life of individuals living with dementia and caregivers and the far-reaching societal consequences. Caregivers managing BPSD experience reduced employment and income, higher out-of-pocket costs, higher rates of mental and physical health issues, and increased subjective burden [5] [6]. Additionally, the COVID-19 pandemic has created unprecedented caregiver challenges, including the disruption of stable routines, increased precautions for regular mask-wearing and hand-washing, reduction or discontinuation of services and supports for both people living with dementia and their caregivers, and a reported increase in BPSD and social isolation [7] [8]. These circumstances have led to a greater reliance on remote, online platforms to support families in caring for people living with dementia as well as support for caregivers themselves [8] [9]. Specifically, the unique and wide-ranging support needs among dementia caregivers and individuals (dyads) highlight the need for novel, person-centered, web-based interventions for caregivers managing BPSD [10] [11] [12].

Non-pharmacological interventions such as online applications have not been rigorously tested. The few that have, have been shown to be safe and provide much-needed support to caregivers [13]. A leading example is the web-based tool, the WeCareAdvisor (WCA). WCA tailors user messaging based on contextual input from a family caregiver about the person living with dementia, their living environment and behavioral challenges. By identifying and monitoring ongoing BPSD, the WCA offers iterative support and, in a pilot randomized trial, was shown to decrease distress and enhance confidence after one month of use [12].

Originally, the WCA was accessible only through a preloaded, self-contained iPad application provided to caregivers. Additionally, automated motivational tips were generated by the application and regularly emailed to participants during use of the program, as well as a compendium of information on dementia contained within the tool (“Caregiver Survival Guide”). Building on these findings, the tool is undergoing modifications to respond to feedback gathered during the pilot phase to enhance the utility of the application. The WCA redesign addresses accessibility considerations by enabling the online platform to be available on common devices (e.g., smartphone, tablet, desktop computer), allowing for ease of functionality and security protections for end users. Authenticated distribution on iOS, Android, and Web platforms will permit future platform updates and secure access to the WCA tool. Additionally, messaging and reminder options can be customized to individual caregiver preferences. Lastly, text-to-speech translation will be available for select modules within the WCA tool to provide expanded accessibility options for a wider caregiver audience. The planned redesign is intended to improve the overall user experience through enhanced interactivity, ease of use, and security. A phase 3 clinical trial of the WCA tool is scheduled to launch in Spring of 2021 [14].

Clinical trials are necessary to ensure the efficacy of burgeoning online resources for people living with dementia and their caregivers living in the community setting. As such, a large, representative sample of caregivers managing BPSD must be identified and recruited to test the utility of the redesigned WeCareAdvisor application. To avoid high costs, study delays, and low-quality evidence, recruitment needs to be conducted efficiently to enroll individuals in a timely manner [15] [16]. Culturally adapted recruitment strategies that reflect the diverse values, beliefs and perceptions of caregiving are crucial considerations to encourage study-participation among caregivers [17]. The WCA trial seeks to recruit and enroll a racially, ethnically, geographically, and socioeconomically diverse sample to examine intervention benefits for diverse caregivers and increase generalizability of study outcomes [15].

Geographic Information Systems (GIS) provide a cost-effective and efficient method to identify individuals of varying demographics, including race and ethnicity, age, gender, geography, urbanicity, and socioeconomic status [16]. This is possible through importing United States Census data, which are freely available online, into GIS software to create maps and inform the targeted distribution of recruitment materials and outreach efforts [18] [19]. Evidence shows that GIS analysis is a cost-effective means of identifying and recruiting racially and ethnically diverse populations for research studies. GIS analysis can also be coupled with partnerships with trusted local community resources serving targeted populations [20] [21]. Sharing recruitment materials with specific community resources may increase awareness and interest among diverse populations of caregivers who could benefit from non-pharmacological interventions but who may not have access to needed supportive services [15].

GIS have been used in research for several areas of inquiry. These include analyzing disease hot spots to identify potential disparities [21], targeting of specific populations for recruitment or analysis of built environment characteristics [19] [22], and racial disparities in access to healthcare [23]. GIS have also been used to identify at risk adult populations in community settings [24], predict patterns of chronic conditions common in older populations [25], and compare neighborhood-level analyses (e.g., walkability, green spaces) to older adult health outcomes [26] [27].

As to dementia care, there is limited research on the use of GIS to recruit study participants in trials. In a previous trial, we showed that GIS could be a useful tool by demonstrating retrospectively that GIS strategies were able to predict zipcodes of study participants [16]. However, GIS have not been used to prospectively guide recruitment strategies to identify study participation of caregivers of people living with dementia nationally in a clinical trial. Leveraging the ability to geographically identify organizations and service providers for caregivers managing BPSD may enhance efficiency and outreach of recruitment efforts for nonpharmacological trials. Subsequently, this may result in more diverse study samples in terms of race, ethnicity, socioeconomic status, and geographic locations. This article describes our approach for recruiting caregivers nationwide using GIS methodology to guide outreach. We describe the protocol we plan to follow using GIS and discuss its implications for developing targeted recruitment activities for clinical trials in dementia care.

## 2. Methods

### 2.1. Overview of Study Design and Procedures

The WeCareAdvisor study is a Phase III clinical trial to evaluate the efficacy of this novel, online platform to reduce distress and enhance confidence in caregivers and reduce the occurrences of BPSD in people living with dementia.

To participate in this trial, caregivers must identify as being the primary caregiver of a person living with dementia for at least six months, be 21 years of age or older, speak and read English, and own an internet-capable device they are comfortable using. Additionally, they must not have visual or hearing impairments that affect their use of the WCA or participation in telephone interviews, although use of accommodating devices would be permissible. They also must not be currently enrolled in another caregiver education, support or behavior study. The person they care for must live in the community with no plans to move to a long-term-care facility within the next six months, have at least one BPSD, be able to respond to their surroundings, be able to bring food to their mouths, and be on a stable dose of anti-dementia or psychotropic medications (if prescribed). Within two days of the baseline interview, caregivers will be randomly assigned to one of four groups: 1) immediate use of the WCA and receipt of high-intensity prompts (weekly email or text and telephone call reminders to use the platform), 2) immediate use of the WCA and receipt of a weekly email reminder to use the platform, 3) 3-month delayed waitlist control group receiving high-intensity prompts once they start using WCA, or 4) 3-month delayed waitlist control group with low-intensity reminders once they start using WCA. Caregivers who are placed in the first two groups to immediately begin use of the WCA will receive training in its use and will be encouraged to use it for six months. Caregivers who are assigned to a waitlist control group will receive access to the WCA after a three-month waiting period and will be encouraged to use the WCA for 3 months. After the initial baseline interview, all caregivers will complete three more phone interviews at one, three, and six months. Upon completion of the six-month interview, caregivers will no longer have access to the WCA tool, but will be provided a book with many of the strategies found in the WCA [28]. Finally, caregivers will complete a post-study survey about their experiences participating in this study and using the WCA.

### 2.2. Recruitment Plan

Based on power analysis, a total of 326 caregivers will be enrolled from the 48 contiguous United States and Washington D.C. by the Drexel University research site. The GIS strategy we have developed will compliment and extend three primary recruitment strategies successfully used in previous caregiver studies: 1) collaboration with local and national aging, dementia and caregiver services (e.g., area agencies on aging, adult day sites, senior centers) to send targeted recruitment materials about the study through their service lists; 2) novel outreach strategies including IRB-approved advertising through social media (Facebook, Reddit, Twitter), postings and presentations to community settings (religious organizations, caregiver support groups); and 3) snowball recruitment in which enrolled participants help to identify prospective participants.

### 2.3. GIS Recruitment Protocol

**Figure 1.**
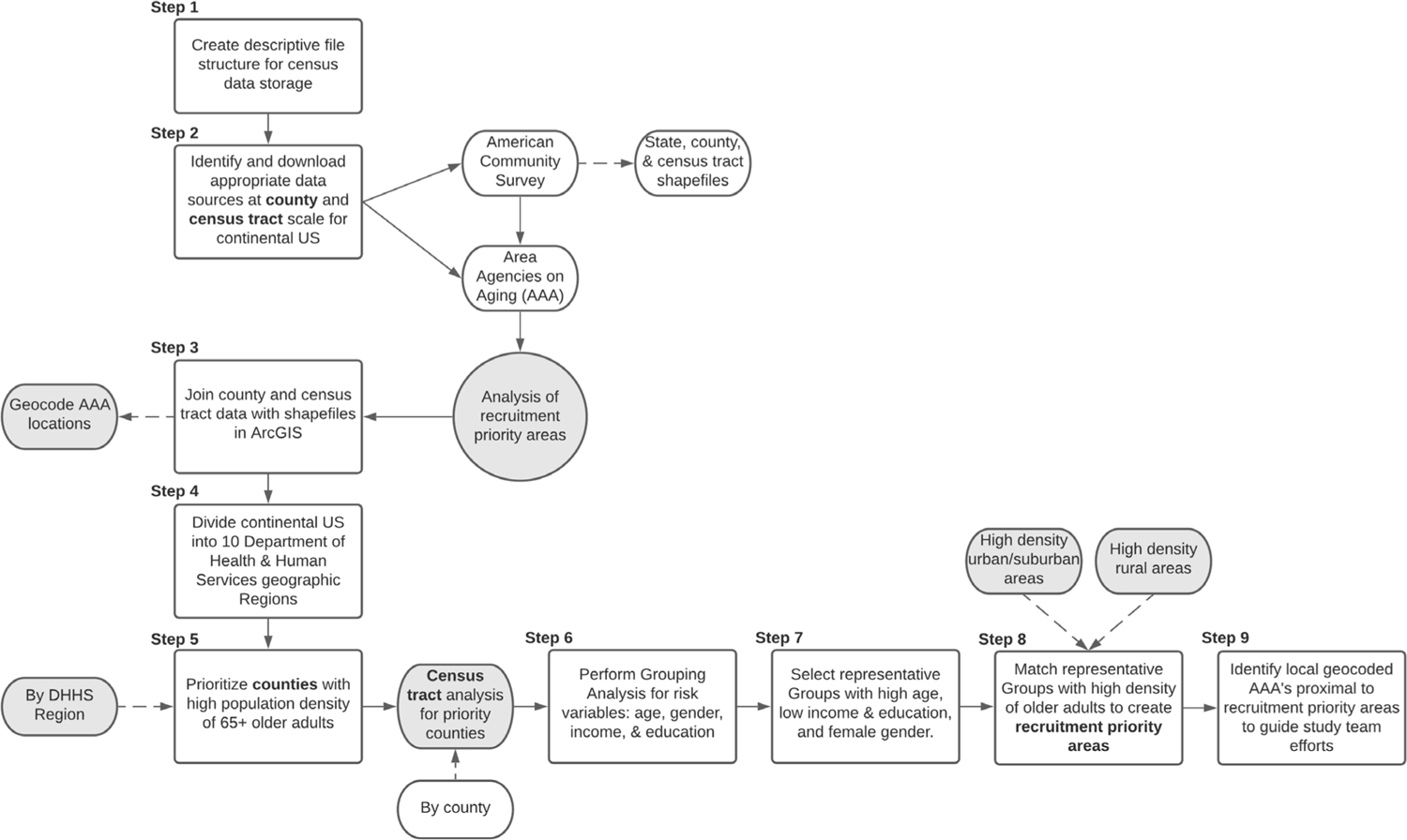
Illustrated workflow for the WeCare GIS recruitment protocol.

In using GIS as an approach to develop a recruitment plan, a nine-step workflow will be followed as described below and illustrated in Figure 1.

#### Step 1. Creation of a file structure for data storage

Due to the extensive geographic area being leveraged for this project, a comprehensive data organizational structure will be created. This file structure features individual folders for each of the 48 contiguous United States and Washington D.C. with sub folders containing county-level data and census tract level data, respectively. Descriptive naming will be used to label data according to the data source, the year of the extract, and the data type.

#### Step 2. Identification and download of appropriate data sources for GIS protocol

##### Geographic data:

The American Community Survey (ACS) is conducted by the U.S. Census Bureau each month in a random, geographically dispersed sample of U.S. households and collects data on education, housing, jobs, commute times, computer/internet usage, disability status, household membership and more. The results of the ACS are used by government and non-government entities to make decisions such as where to construct schools, medical care facilities, and other needed infrastructure [29] [30]. One question assesses the familial relationship of others residing in the home to the householder completing the survey [30]. Primary data for this analysis will be collected from georeferenced digital layers at the county and census tract spatial scales which are publicly available from the U.S. Census Bureau in the 2018 and 2019 ACS 1-year and 5-year estimates. National analyses will be performed in sequence from the county-level to the census tract level in order to broadly identify geographic patterns of interest leading to more local, granular investigation. The study area will include the 48 contiguous United States and Washington DC. At the county level, available data will be limited to select counties with a population greater than or equal to 65,000 included in the 2019 ACS 1-year data estimates and include 748 counties with populations ranging from 65,055 to 10,039,107. Data downloaded for analysis at this level will include age and gender. The 1-year estimates reflect the most recently collected data available and are useful for geographic areas with population characteristics that are expected to change rapidly. The 2018 ACS 5-year estimates will be used for census tract level analyses due to smaller margins of error compared to 1-year data, increased statistical reliability for this geographic scale, and complete data for all areas [31]. Datasets will be downloaded summarizing estimates of age, gender, median household income, and education levels for all census tracts in 48 states and the District of Columbia. The study area at the census tract level will include 72,539 census tracts.

##### Regional organizational resources:

Area Agencies on Aging (AAA), established by the Older Americans Act of 1973, are charged with planning, developing, coordinating, and delivering a range of care services for older adults. Meals, transportation, legal services, caregiver support, and health care resource referral are among the services provided by AAAs. The number of AAAs varies from state to state and is determined by bureaucratic divisions called Planning and Service Areas. While some services such as legal aid are standard, most programs offered by each AAA are informed by the needs and available services in the surrounding community. Given this diversity, AAA offerings in some states are minimal, whereas others are numerous and offer evidence-based interventions for caregivers. Through planning and coordination with local service providers, AAAs are an invaluable resource and source of information for older adults and their caregivers [32]. As a geographic center for services and information, AAAs are important partners for study recruitment.

Address, phone, email, web address, geographic area served, and appropriate contact information for nationwide AAAs will be downloaded from the official agency-hosted websites and stored in a consolidated database for geocoding in ArcGIS. 733 AAAs across all 50 states and Washington D.C. will be collected and geocoded for analysis in this recruitment model. These 733 agencies serve, to the best of the authors’ knowledge, all Planning and Service Areas in the United States.

#### Step 3. Joining data in ArcGIS

Downloaded census data will be imported into ESRI ArcGIS v. 10.7.1. All data will then be matched to the corresponding geographic reference level in ArcGIS and joined to matching shapefiles based on a 14-digit GEOID code. GEOIDs facilitate the organization, presentation, and sharing of statistical spatial data, and are crucial for interpolating demographic to geographic data. This code is used to identify the summary level of the data, the geographic component (*i.e.*, county, ZIP, census tract), and the Federal Information Processing Standards code which uniquely identifies local information such as the state [33]. Area Agencies on Aging will be geocoded by physical address and added to the recruitment map as a separate layer for further analysis.

#### Step 4. Organization of national geography

To focus recruitment efforts, data will be organized according to the U.S. Department of Health & Human Services (DHHS) geographic regions within the GIS analysis. The Office of Intergovernmental and External Affairs has established 10 Regional Offices that directly serve local state organizations to address the needs of communities served through DHHS policies and programs [34]. These regions consist of 4 – 8 states, each grouped in close geographic proximity. For the purposes of this project, the DHHS regions provide a proven organization template to define distinct areas for analysis.

#### Step 5. National prioritization of high-density countries

Geographic regions will first be analyzed individually at the county level to guide subsequent in-depth analysis at the local, census tract reference level. Population density of adults 65 years of age and above will be calculated for each county with available data in each region. The density of older adults in each county will be compared to the region average, and those within two standard deviations (SD) above the average (−0.5 to 1.5 SD range) will be selected and isolated for in-depth analysis at the census tract level. These steps will be repeated to compare the population density of each county with the state average to check for significant differences based on state vs. total region density averages. These steps will be repeated for population density of individuals 65 years old and above filtered by specific race and gender characteristics.

#### Step 6. Perform grouping analysis

Recruitment priority areas will be defined at the census tract geographic data level to detect more precise areas for action. Individuals at risk of either having dementia or being a caregiver of a person living with dementia can be described by specific risk variables according to previous studies [1] [35] [36] [37] [38]. The variables for age, gender, income, and education level will be utilized for this recruitment analysis based on previous research and verified in retrospective work done by Scerpella *et al.* (2019) [16]. A grouping analytical approach will be used to derive optimal numbers of high and low prevalence tracts wherein specific combination of these critical variables can be observed. The Grouping Analysis tool in ArcGIS stratifies variables in each census tract into groups so that all the variables included within each resulting group are as similar as possible (e.g., grouping high age and high prevalence of women), while each group is as different from the other generated groupings as possible. The number of groups created using the Grouping Analysis tool is user-defined. The total number of groups is verified by referencing a pseudo F-statistic, ensuring a statistically meaningful number of output groups for each stratified variable [39].

#### Step 7. Selecting representative groups based on prevalence of risk variables

Analyzing the resulting groups generated by the Grouping Analysis tool, those representing the global upper quartile for age and gender and the global lower quartile for income and education will be selected. The census tract polygons that will be identified using this procedure will be further analyzed to create recruitment priority areas.

#### Step 8. Match selected groups with high population density areas for individuals aged 65+

The population density of individuals 65 years old and above will be verified for the census tracts selected during the Grouping Analysis phase to account for the effects of geographic distribution on recruitment areas. Our previous research using GIS determined that this age cut off maximizes the potential for identifying households in which a family member is a caregiver of a person living with dementia [16]. Comparing the total density of older adults in each tract to the state average, tracts with a density −0.5 to 1.5 SD of the state average will be selected using the overlay selection tool and a 0.5-mile Euclidian distance spatial buffer applied to the tract geometry. The spatial buffer increases the representative area of a polygon in all directions to account for the reduced mobility of older adults in the U.S. and an inverse relationship between age and travel outside of a half-mile [40] [41].

The resulting recruitment priority areas are based on both risk variables for this population as well as population density. These locations reflect a national representation of potential locations in which community-dwelling people living with dementia and their caregivers may reside. The density considerations described thus far do not account for potentially overlooked populations of older adults living in low density areas such as rural or suburban communities. Mirroring the density and buffer procedures above, census tracts with a population density of people 65 and over that is less than −0.5 SD from the state average will be separated for deeper inspection. Analyzing only these less dense regions, the census tracts with a population density of older adults up to 2 SD above the mean will be highlighted and buffered. While these recruitment areas are not likely to yield large numbers of participants for research, understanding actionable locations in less-represented areas potentially allows for targeted recruitment of traditionally geographically isolated older adults and their caregivers.

#### Step 9. Compare recruitment priority areas to geocoded AAAs to guide recruitment methods

AAAs represent a resource that serves older adults and are located across the continental United States. Comparing the proximity (near and far) of recruitment priority areas to the geocoded locations of these agencies will allow for outreach to representative participants while establishing these centers as local reference points within potentially actionable zones. The creation of this new infrastructure will be utilized to test multiple, co-occurring recruitment strategies. Possible strategies include sending targeted mailings to AAAs proximate to recruitment priority areas, focusing on areas that are underserved by AAAs, or targeting online advertising by zip codes or other relevant geographic identifiers. Testing and utilizing multiple recruitment strategies with a national database provides a more rapid learning system to recruit and enroll more caregivers quickly, reducing the overall time it takes to evaluate interventions and publish randomized clinical trial findings [42]. In conjunction with this GIS protocol, it is hoped that these strategies will be employed where they will have the most efficacy.

## 3. Expected Results

It is anticipated that this 9-step process will result in a diverse sample for the WCA trial in terms of geographic, race, ethnic, and socioeconomic representation. Our approach is grounded in a previous study that informs our approach here [16]. In a retrospective review of enrollment for a previous dementia care trial, we found that priority areas devised using GIS matched the actual locations of study participants. That is, we found that 89% of study participants resided within a defined recruitment priority area within urban/suburban localities identified via GIS. Furthermore, 28% of participants were identified within recruitment priority areas that were created to identify rural communities. Based on results from this previous retrospective approach, we anticipate that we will be able to proactively identify specific geographic areas of high density of people living with dementia and their caregivers.

## 4. Discussion

The impacts of the COVID-19 pandemic continue to force widespread restructuring of practices across many industries, including research. The challenges associated with this unprecedented global crisis helped to emphasize the continuing public health priority to improve support services for community-dwelling people living with dementia and their caregivers. In response to mandated social distancing safety precautions, many industries have been forced to employ new, remote technologies. Given the changing research landscape and uncertainty about future in-person research activities, the WCA clinical trial has shifted gears from a local in-person recruitment and interviewing approach to national outreach and telephonic participation. This has afforded a unique opportunity to rethink recruitment strategies to obtain a more representative national sample of caregivers. Foregoing repeated in-home participant interviews, assessments, and training, study activities have been modified to be conducted remotely for the safety of both participants and the study team. This also has included adjusting protocols and interview methodologies to be suitable for remote administration.

Technology-based interventions provide opportunities to include a broader sample of oft-overlooked participants to both benefit from resources that are generally unavailable and to strengthen the generalizability of study findings [43]. However, the inability to directly interact with individuals presents several challenges for enrollment. While online recruitment initiatives can be a successful means of reaching potential participants, the results are inconsistent and unreliable when specifically targeting older adults and caregivers and may not be representative nor include Black, Brown, Latinx or other race/ethnic groups [44] [45]. Not enough research has been conducted to fully understand this limitation, but the literature suggests that while no singularly effective strategy stands out, utilizing a variety of novel and unique strategies is critical to reaching recruitment goals and diversifying samples [46]. GIS provide the tools to extend outreach ng while leveraging key local resources and customizing recruitment messaging to cultural preferences and values.

While most research using GIS focus on hyperlocal contexts, the current protocol seeks to build a GIS database on a national scale with the flexibility to identify potentially high-yield areas for recruitment based on flexible variables of interest. Building on retrospective work showing the effectiveness of GIS to locate a dementia sample for a nonpharmacological clinical trial [16], the protocol proposed herein is designed to prospectively identify locations nationally where older adults with dementia and their caregivers reside. This is based on freely available census data and careful consideration of local aging agencies. By strategically targeting local community centers to serve as recruitment touchstones within priority areas across the ten predetermined DHHS regions, the study can increase the generalizability of its results. It also enables recruitment of representative samples to address known systemic health disparities resulting from health access inequities and imprecisely tested evidence-based treatments.

While recruitment challenges will inevitably persist, GIS enables reasonably informed decision making to allow for emergent exploration of local circumstances based on continuous recruitment innovation and stakeholder feedback. This adaptive, iterative strategy lends itself to a rapid research learning systems approach in the field of health research. This, in turn, allows data from community stakeholders, healthcare systems, and care networks to be synthesized to produce swift innovation in research methodologies [42]. Employing a diversity of services and recruitment tactics, the WCA study aims to reduce extraneous and potentially costly efforts for identifying characteristic participants by highlighting geographic areas with high concentrations of desired participants and avoiding areas that are unlikely to host relevant individuals. This protocol details methodologies for identifying service providers located in demographically rich populations that may serve as study partners. With these partners, it may be possible to achieve greater community engagement and capacity to bolster recruitment efforts and outcomes. Study staff must continue to develop culturally relevant recruitment materials and engage with community organizations and community members that they aim to enroll into their studies [21]. As ongoing recruitment efforts continue, this GIS methodology will allow researchers the flexibility to expand or filter additional information. Based on evolving priorities such as race or data pertaining to the built neighborhood environment, it may be possible to meaningfully diversify the enrollment population. Rural communities are frequently excluded from studies due to location, convenience sampling, and difficulty for residents to access community information. A diverse body of participants is necessary to ensure that the intervention is effective for all intended caregivers [47]. It is also beneficial to identify types of adaptations needed for recruitment of specific demographic groups and this in turn can help inform widespread dissemination of WCA tool if found efficacious in the trial [48].

There are several limitations to the protocol detailed in this manuscript. Census data used for this project consist of multi-year estimates extrapolated from data collected during the 2010 U.S. Census. Due to population mobility during the last decade, the estimates used for this analysis may not be wholly representative of true residency patterns of older adults. As noted in the description of methods, ACS Census estimates at the county-level are selective and do not represent areas with a population less than 65,000. While the protocol attempts to control for this at the census tract level, national identification of priority areas for analysis is limited to the county data available for download and may therefore neglect the most remote populations. Another limitation of the study concerns complications related to the complexity of performing remote recruitment efforts, primarily including postal mailings and partnering with local centers and agencies. This protocol aims to address both limitations by increasing the precision of these efforts to reduce wasted time and resources. However, challenges may endure.

## 5. Conclusions

Overall, the current protocol depicts a methodology to predetermine geographic locations containing high concentrations of potential participants of interest to this study, caregivers, as well as local resources that may have pre-established connections to this population. Future directions for this project include actively tracking, analyzing, and adjusting the sensitivity of the GIS methodologies. This may be done by mapping the location of enrolled participants during the study recruitment phase and comparing these to the predicted catchment areas. Comparing predetermined recruitment areas with the locations of actual enrollment will help improve the prognostic ability of this model. The current study intends to employ a diverse array of strategies for recruitment. By carefully tracking recruitment methods, successfully enrolled participants can be analyzed within their geographic context to determine local and demographic variables that may inform efforts in similar communities elsewhere in the nation. The scope of this geographic recruitment model lends itself to broad applicability across future research projects. The methods detailed here allow not only for the prioritization of factors which pertain to a population of people living with dementia and their caregivers, but also for vulnerability measures unique to a range of community-dwelling, representative participants sought for recruitment into a variety of clinical trials.

If proven, this GIS model for recruitment could be used to more precisely connect researchers and caregivers through partnerships with local resources and stakeholders in communities of interest. It will also aid in the development of a national database of potential locations of people with dementia and their caregivers and facilitate the ability to test multiple recruitment strategies at once.

## Nomenclature

AAA: Area Agencies on Aging
ACS: American Community Survey
BPSD: Behavioral and Psychological Symptoms of Dementia
DHHS: Department of Health & Human Services
GIS: Geographic Information Systems
PLWD: People living with dementia
SD: Standard Deviation
WCA: We Care Advisor
